# The Prognostic Power of miR-21 in Breast Cancer: A Systematic Review and Meta-Analysis

**DOI:** 10.3390/ijms26199713

**Published:** 2025-10-06

**Authors:** Luana Conte, Maria Rosaria Tumolo, Giorgio De Nunzio, Ugo De Giorgi, Roberto Guarino, Donato Cascio, Federico Cucci

**Affiliations:** 1 Department of Physics and Chemistry “E. Segrè”, University of Palermo, 90128 Palermo, Italy; luana.conte@unipa.it (L.C.); donato.cascio@unipa.it (D.C.); 2 Department of Biological & Environmental Sciences & Technology, University of Salento, 73100 Lecce, Italy; 3 Research Unit of Brindisi, National Research Council, Institute for Research on Population & Social Policies, 72100 Brindisi, Italy; 4 Laboratory of Biomedical Physics and Environment, Department of Mathematics and Physics “E. De Giorgi”, University of Salento, 73100 Lecce, Italy; giorgio.denunzio@unisalento.it; 5 Advanced Data Analysis in Medicine (ADAM), Laboratory of Interdisciplinary Research Applied to Medicine (DReAM), Local Health Authority (ASL) of Lecce, 73100 Lecce, Italy; 6 Department of Experimental Medicine, University of Salento, 73100 Lecce, Italy; ugo.degiorgi@unisalento.it; 7 Branch of Lecce, National Research Council, Institute of Clinical Physiology, 73100 Lecce, Italy; roberto.guarino@cnr.it; 8 Gruppo Villa Maria, Città di Lecce Hospital, 73100 Lecce, Italy; fcucci@gvmnet.it

**Keywords:** miR-21, breast cancer, prognosis, meta-analysis, bioinformatic

## Abstract

Breast cancer (BC) is one of the most common malignancies among women worldwide. Despite advances in early detection and treatment, prognosis remains highly variable. Molecular biomarkers, such as microRNAs (miRNAs), have emerged as promising tools to refine prognostic assessment. Among them, miR-21 is consistently overexpressed in solid tumors and implicated in key oncogenic pathways. This systematic review and meta-analysis aimed to clarify the prognostic significance of miR-21 in BC and explore its molecular mechanisms through bioinformatic analyses. A systematic search of PubMed, Scopus, and Web of Science up to April 2025 identified 18 eligible observational studies. Pooled analyses showed that high miR-21 expression was significantly associated with poorer overall survival (OS) (HR = 2.37, 95% CI: 1.42–3.98) and recurrence-related outcomes (DFS/RFS) (HR = 2.10, 95% CI: 1.32–3.34). Subgroup analyses confirmed robust associations across different cut-off definitions and revealed particularly strong effects in triple-negative BC (HR = 5.69) and mixed subtypes (HR = 2.55), but no significant association in HER2-positive BC. Bioinformatic analysis identified target genes such as PTEN, BCL2, STAT3, and MYC, involved in apoptosis regulation, proliferation, NF-κB signaling, and immune modulation. These findings provide consistent evidence that miR-21 is a promising minimally invasive prognostic biomarker in BC, particularly in aggressive subtypes, and support its integration into future multimodal prognostic models.

## 1. Introduction

Breast cancer (BC) remains the most prevalent malignancy among women worldwide and represents a major public health challenge. In 2022, approximately 2.3 million new cases and 670,000 related deaths were recorded globally, accounting for nearly one-third of all female cancers [1]. Projections estimate that by 2040, annual incidence will exceed 3 million cases, with mortality approaching 1 million deaths, underscoring the pressing need for improved disease management strategies [2].

Despite significant advances in early detection and treatment, prognosis remains highly variable due to a range of clinical and pathological factors [3,4,5]. Key prognostic indicators include tumor size, lymph node involvement, histological grade, and molecular subtype [6,7,8]. According to recent global estimates, the 5-year relative survival rate exceeds 90% for patients with localized disease but decreases substantially in cases with regional lymph node involvement or distant metastases [9,10].

Patients with hormone receptor-positive, human epidermal growth factor receptor-2 (HER2)-negative tumors tend to have a more favorable prognosis, whereas those with triple-negative or HER2-positive subtypes typically exhibit more aggressive clinical behavior and poorer survival outcomes [11,12].

These observations highlight the urgent need for robust and accessible prognostic biomarkers that can integrate with current prognostic models to improve risk stratification, guide treatment decisions, and follow-up strategies, toward a more personalized oncology [13,14,15].

In recent years, attention has shifted toward the identification of molecular biomarkers capable of refining prognostic assessments beyond traditional clinicopathological parameters. Among these, microRNAs (miRNAs), small, non-coding RNA molecules that regulate gene expression post-transcriptionally, have emerged as promising candidates due to their stability in biofluids and their involvement in key oncogenic and tumor suppressor pathways [16,17,18]. Dysregulated expression of specific miRNAs has been observed across various malignancies, where they can function as oncogenes (oncomiRs) or tumor suppressors. OncomiRs are typically overexpressed in tumors and downregulate tumor suppressor genes [19], while tumor-suppressive miRNAs are often silenced in cancer and target oncogenic pathways [20].

MiR-21 stands out as one of the most consistently overexpressed miRNAs in solid tumors, including BC [21,22]. It contributes to tumor progression by targeting multiple tumor suppressor genes such as PTEN and PDCD4, which in turn modules key oncogenic pathways including PI3K/AKT and MAPK/ERK, thereby promoting cell survival, invasion, and metastatic potential [23,24,25].

Elevated levels of miR-21, detectable in tissue, serum, plasma, etc., have been associated with more aggressive disease and poorer survival outcomes in several studies [26,27].

Given its oncogenic role and detectability in circulating biofluids, miR-21 has attracted growing interest as a potential prognostic biomarker in BC. Numerous observational studies have investigated its association with survival outcomes, but the evidence to date remains ambiguous: while some reports suggest that high miR-21 expression predicts poor prognosis, others found no significant association [28,29]. These apparent discrepancies may reflect differences in study design, sample type, analytical methods, or thresholds used to define expression levels.

Several systematic reviews and meta-analyses have already explored the role of miR-21 in BC. For instance, Wang et al. (2023) examined the diagnostic and predictive value of exosome-derived miR-21 from liquid biopsy samples, highlighting its promise as a minimally invasive biomarker [26]. Earlier meta-analyses also evaluated miR-21 either in combination with other miRNAs or in specific subtypes such as triple-negative BC (TNBC) [30]. However, these works either emphasized diagnostic rather than prognostic outcomes, restricted their scope to selected BC populations, or did not incorporate the most recent studies.

In contrast, the present systematic review and meta-analysis focuses exclusively on the prognostic value of miR-21 across all BC subtypes. By updating the evidence base with the most recent literature and providing a quantitative synthesis with subgroup and sensitivity analyses, we aim to clarify the prognostic significance of this key oncomiR in BC.

## 2. Materials and Methods

### 2.1. Protocol and Registration

This systematic review was conducted in accordance with the Preferred Reporting Items for Systematic Reviews and Meta-Analyses (PRISMA) 2020 guidelines and registered on the Open Science Framework (OSF) platform (https://doi.org/10.17605/OSF.IO/ZVA2C, accessed on 4 May 2025) to ensure transparency and reproducibility. The PRISMA 2020 checklist can be found in Supplementary Materials (Table S1).

### 2.2. Eligibility Criteria

Studies were eligible for inclusion if they met the following criteria: (i) examined the association between miR-21 expression and survival outcomes in patients with BC; (ii) reported or provide sufficient data to calculate hazard ratios (HRs) with 95% confidence intervals (CIs) for Overall Survival (OS), Disease-Free Survival (DFS), Recurrence-Free Survival (RFS), or Disease-Free Interval (DFI); and (iii) analyzed human tissue, serum, plasma, or other biological fluids.

The following were excluded: review articles, commentaries, letters, case reports, or conference abstracts without full data; duplicates/overlapping datasets (only the most comprehensive study was retained); non-human or purely in vitro/preclinical studies; global miRNA-profiling reports without miR-21–specific results; studies in which miR-21 was included only as part of a multi-miRNA prognostic signature/model without separate analysis; studies that reported results that were incomplete, inconclusive, or not stratified for miR-21 expression.

Table 1 shows the Population-Prognostic Factors-Outcome (PFO) framework used to clearly define the search strategy.

### 2.3. Information Sources and Search Strategy

PubMed, Scopus, and Web of Science (WOS) were systematically searched up to April 2025.

Two reviewers (L.C and F.C) developed search strings based on the PFO framework, combining free-text terms and controlled vocabulary in the title and abstract fields. Boolean operators (AND, OR) were used to link key concepts related to ‘miRNA-21’, ‘breast’, ‘cancer’, and ‘expression terms’, to capture studies evaluating miR-21 expression levels and their prognostic performance. The final strategies were approved by the research team. Full details are provided in Supplementary Table S1.

### 2.4. Selection Process, Data Collection Process and Data Items

Three reviewers (L.C., M.R.T. and F.C) independently screened titles and abstracts and subsequently assessed the full texts of potentially eligible studies. Disagreements were resolved by discussion and consensus. Reference lists of included studies were also manually searched to identify additional eligible studies.

The same three reviewers (L.C., M.R.T., and F.C.) independently extracted data from the included studies using a standardized form. Any discrepancies were resolved by discussion and consensus. When necessary, study authors were contacted to obtain clarification or missing information.

The following data items were collected from each study: first author, year of publication, study design, sample size, sample source, disease stage, miR-21 detection method, cut-off definition for high vs. low expression, survival outcomes assessed (OS, DFS, RFS, DFI), follow-up duration, and reported HRs with 95% CI.

### 2.5. Quality Assessment

Methodological quality was assessed using the Dutch Cochrane Centre checklist, as recommended by the MOOSE guidelines [31]. The checklist was used to evaluate key aspects such as population and setting, study design and outcome assessment, miR-21 measurement technique and cut-off definition, survival outcomes reported, follow-up duration, and country of origin. Minor missing information (e.g., follow-up not explicitly reported) did not lead to automatic exclusion if sufficient outcome data were available for the meta-analysis.

### 2.6. Statistical Methods

For each included study, HRs and 95% CIs were extracted or computed. Heterogeneity was assessed using Cochran’s Q test and the Higgins I^2^ statistic. A statistically significant heterogeneity was defined by a *p*-value less than 0.05 and/or an I^2^ greater than 50% [32]. When heterogeneity was present, a random-effects model was applied using the DerSimonian-Laird method [33]; otherwise, a fixed-effect model was used according to the Mantel-Haenszel method [34].

Forest plots were generated to visually represent the aggregated effect of miR-21 expression on survival outcomes, including OS and DFS/RFS. An HR > 1 signified poorer prognosis with high miR-21 expression and was deemed significant when its 95% CI excluded 1. Publication bias was evaluated by funnel plots inspection and Egger’s test (*p* > 0.05 implying no significant bias). Sensitivity analyses were performed by sequentially omitting each study.

To further explore heterogeneity, subgroup analyses were conducted by sample source, disease stage, and ethnicity. Subgroup-specific pooled HRs were computed using the same fixed/random-effects rules as in the primary analysis; pooling was undertaken only when ≥2 studies were available for a given stratum.

All analyses were conducted in Review Manager version 5.1 (The Cochrane Collaboration, Oxford, United Kingdom) and Python version 3.11.

## 3. Results

### 3.1. Literature Search

A total of 874 records were identified through database searches, including 577 from PubMed, 215 from Scopus, and 82 from WOS. No additional records were retrieved from other sources or registries. After the removal of 55 duplicates, 819 unique records were assessed.

**Figure 1:**
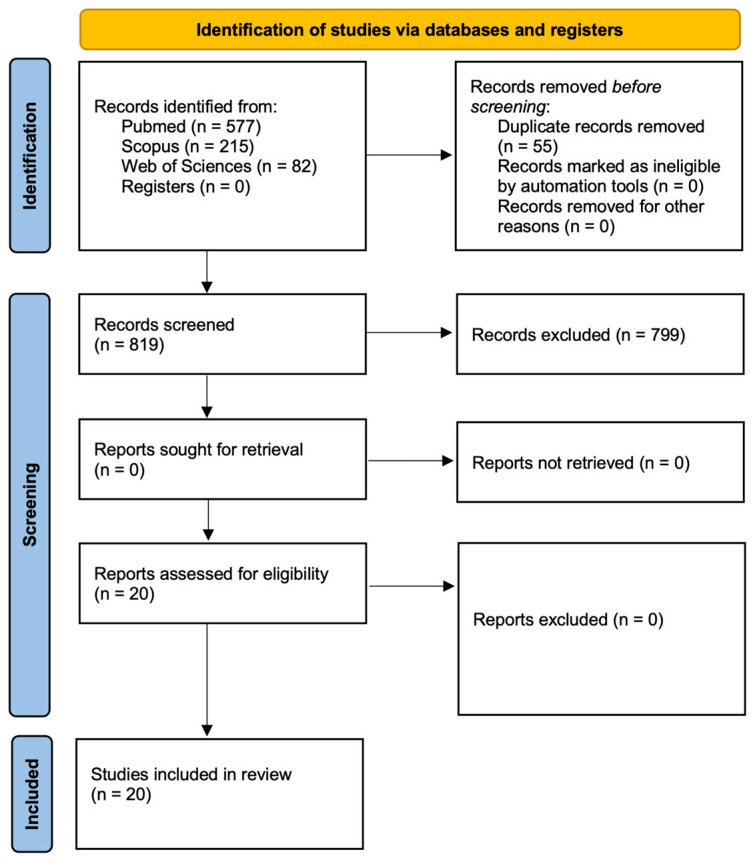
PRISMA 2020 flow diagram of the research.

Following title and abstract screening, 545 articles were excluded for not meeting the eligibility criteria. An additional 254 records were excluded irrespective of content, based on publication type: Book (*n* = 17), Book Chapter (*n* = 8), Meeting Abstract (*n* = 16), Review (*n* = 190), Systematic Review (*n* = 22), Letter (*n* = 1). Ultimately, 20 studies met the inclusion criteria and were retained for final analysis. The study selection process is detailed in Figure 1 according to the PRISMA 2020 flow diagram.

### 3.2. Summary of Included Studies

As depicted in Table 2, the included studies investigated various BC subtypes. Specifically, [35,36,37,38,39] focused on triple-negative BC (TNBC) cases, while [40,41,42] included only HER2-positive patients. The remaining studies analyzed mixed BC populations.

MiR-21 expression was assessed most frequently in tumor tissue (n = 11), followed by serum (n = 5), plasma (n = 3), and bone marrow (n = 1). The cut-off definitions varied: 13 studies used median or mean expression values, whereas others applied fixed thresholds (e.g., 5.84-fold, or 1.5-fold increase).

Regarding survival outcomes, 5 studies reported both OS and DFS [35,42,43,44,45], 8 focused solely on OS [29,37,40,46,47,48,49,50], and 7 evaluated DFS, DFI, and/or RFS [36,38,39,41,51,52,53]. Notably, since [41] reported both DFS and RFS outcomes separately, the study was treated as two distinct entries: [41] for DFS and for RFS.

All included studies met the principal methodological quality criteria of the Dutch Cochrane Centre checklist. In three studies [41,48,53], the follow-up duration was not explicitly reported, and in five studies [38,39,41,42,51] the cut-off value was not specified; nevertheless, these studies were retained as they provided sufficient outcome data to be included in the meta-analysis.

**Table 2:** Clinical characteristics of studies included in the meta-analysis.

First Author, Year, References	Study Design	Method	N Case Group	Sample Source	Stage	Ethnicity	Country	Cut-Off	Survival Analysis	HR (95%CI) for OS	HR (95%CI) for DFS/RFS/DFI	Follow-Up, Months (Range)
Yan, 2008 [29]	Retrospective	RT-qPCR	113	Tumor Tissue	I-III	Asian	China	Mean	OS	4.13 (1.80–9.50)	-	66.2 (10.4–81.0)
Qian, 2009 [46]	Retrospective	RT-qPCR	344	Tumor Tissue	I-IV	Caucasian	USA	Median	OS	0.99 (0.56–1.73)	1.15 (0.69–1.93)	86.2 (8.0–108)
Lee, 2011 [43]	Retrospective	RT-qPCR	109	Tumor Tissue	I-III	Asian	Korea	Mean	OS, DFS	14.21 (1.34–15.10)	0.88 (0.09–8.41)	Median 54
Ota, 2011 [44]	Retrospective	RT-qPCR	291	BM	-	Asian	Japan	5.84	OS, DFS	3.40 (1.26–9.18)	1.04 (0.71–1.48)	61 (2–90)
Walter, 2011 [47]	Retrospective	RT-qPCR	25	Tumor Tissue	I-IV	Caucasian	USA	Median	OS	0.49 (0.06–3.71)	-	Median .5
Radojicic, 2011 [35]	Retrospective	RT-qPCR	49 TNBC	Tumor Tissue	I-IV	Caucasian	Greece	Median	OS, DFS	0.85 (0.09–8.29)	2.49 (0.72–8.58)	120
Markou, 2014 [45]	Retrospective	RT-qPCR	112	Tumor Tissue	I-IV	Caucasian	Greece	Median	OS, DFI	1.48 (0.73–2.98)	1.762 (1.010–3.074)	84 (10–149)
Dong, 2014 [36]	Retrospective	RT-qPCR	72 TNBC	Tumor Tissue	I-IV	Asian	China	1.5	RFS	-	2.32 (1.24–4.12)	95
Muller, 2014 [40]	Retrospective	RT-qPCR	127 HPBC	Serum	I-IV	Caucasian	Germany	Median	OS	5.24 (1.58–17.35)	-	62.15 (5.56–66.28)
Wang, 2015 [41]	Retrospective	RT-qPCR	326 HPBC	Serum	I-III	Asian	China	-	RFS, DFS	-	RFS: 2.942 (1.420–8.325) DFS: 2.732 (1.038–7.273)	-
Yan, 2016 [48]	Retrospective	RT-qPCR	320	Tumor Tissue	I-III	Asian	China	Mean	OS	2.47 (1.08–5.65)	-	-
Liu, 2017 [51]	Prospective	RT-qPCR	118	Serum	II-III	Asian	China	-	DFS	-	51.579 (13.942–190.820)	25 (10–36)
Papadaki, 2018 [52]	Prospective	RT-qPCR	133	Plasma	I-II	Caucasian	Greece	Median	DFS	-	4.557 (1.685–12.869)	94.3 (14.33–159.30)
Papadaki, 2019 [49]	Prospective	RT-qPCR	70 metastatic BC 45 de novo metastatic	Plasma	I-III	Caucasian	Greece	Median	OS	1.589 (0.916–2.756)	-	27.33 (20.97–33.69)
Liu, 2019 [42]	Prospective	RT-qPCR	83 HPBC	Serum	II-III	Asian	China	-	OS, DFS	0.49 (0.21–1.11)	0.51 (0.24–1.08)	Mean 23.6 (13–36)
Wu, 2020 [37]	Retrospective	Sequencing	151 TNBC	Tumor Tissue	II-IV	Asian	China	Median	OS	7.396 (1.590–34.411)	-	60
Romadhon, 2021 [50]	Retrospective	RT-qPCR	49	Plasma	I-II	Asian	Indonesia	Mean	OS	5.5 (3.2–9.46)	-	12
Kujala, 2024 [38]	Prospective	Sequencing	14 recurrent TNBC 19 non recurrent	Serum	II-III	Caucasian	Finland	-	RFS	-	1.87 (1.06–3.30)	60
MacKenzie 2014 [39]	Retrospective	in situ hybridization	901 TNBC	Tumor Tissue	I-II	Caucasian	USA	-	RFS	-	1.71 (1.265–2.319)	median, 10.33 years
Amirfallah 2021 [53]	Retrospective	RT-qPCR	139	Tumor Tissue	I-III	Caucasian	Iceland	Median	DFS	-	1.89 (1.18–3.04)	-

Abbreviations: BC: Breast Cancer; BM: Bone Marrow; DFI: Disease Free Interval; DFS: Disease Free Survival; HPBC: HER2-positive BC; OS: Overall Survival; RFS: recurrence-free survival; TBCN: Triple negative breast cancer.

### 3.3. Correlation Between miR-21 Expression and Overall Survival (OS)

**Figure 2:**
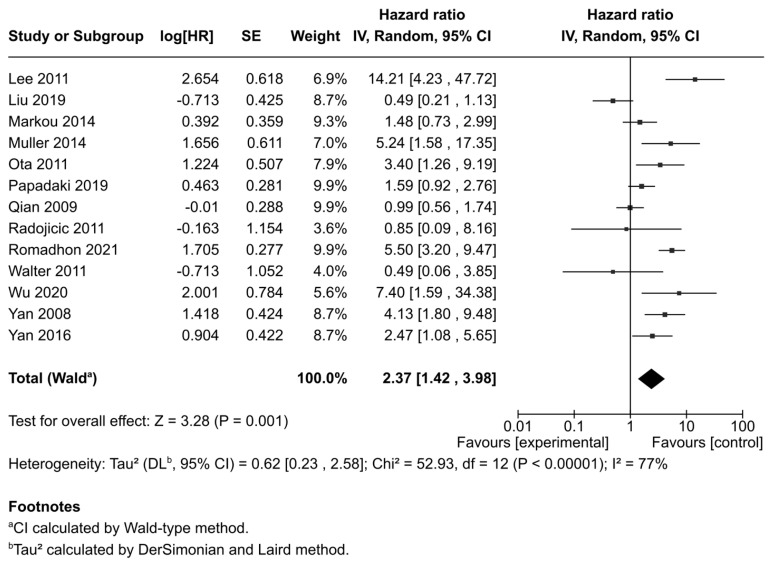
Forest plot showing the association between miR-21 expression and overall survival (OS) across the included studies [29,35,37,40,42,43,44,45,46,47,48,49,50].

Thirteen studies reporting OS were included. Due to significant heterogeneity (I^2^ = 77%, *p* < 0.001), a random-effects model was applied. The pooled analysis demonstrated that high miR-21 expression was significantly associated with poorer OS compared to low expression, with a pooled HR of 2.37 (95% CI: 1.42–3.98; *p* = 0.001) (Figure 2).

### 3.4. Correlation Between miR-21 Expression and Disease-Free Survival/Regression Free Survival

**Figure 3:**
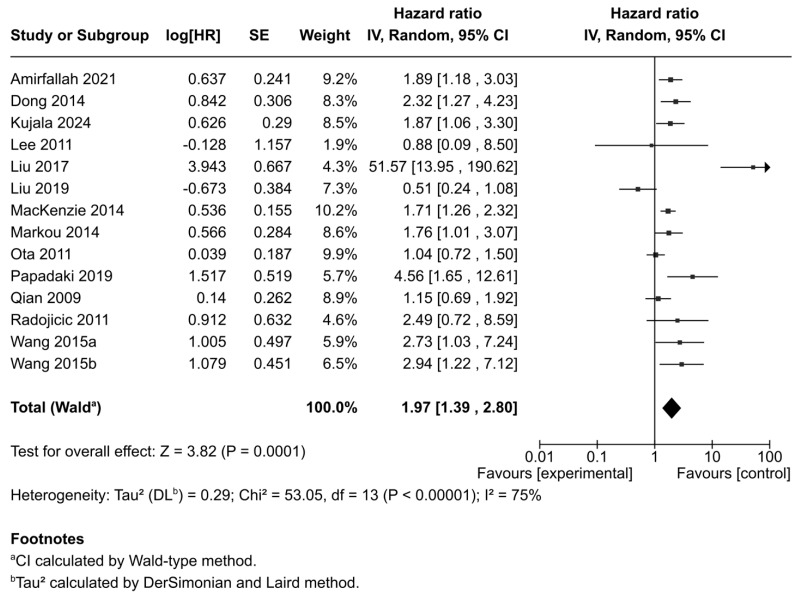
Forest plot showing the association between miR-21 expression and disease-free survival (DFS, DFI, RFS) across the included studies [35,36,38,39,41,42,43,44,45,46,51,52,53].

Thirteen studies reported DFS, DFI, and RFS outcomes. Due to marked heterogeneity (I^2^ = 75%, *p* < 0.001), a random-effects model was again applied. The results revealed that miR-21 overexpression was significantly associated with worse DFS/RFS outcomes in BC patients (HR = 1.97; 95% CI: 1.39–2.80; *p* = 0.0001) (Figure 3).

### 3.5. Subgroup Analysis

To investigate potential sources of heterogeneity, subgroup analyses were performed based on cut-off values for miR-21 overexpression and BC subtype (Table 3).

In the subgroup analysis based on cut-off values, both the mean and median cut-off groups demonstrated a statistically significant association between elevated miR-21 expression and overall survival. When studies used the mean as the cut-off, the pooled HR was 4.81 (95% CI: 3.29–7.02; *p* < 0.001), with moderate heterogeneity (I^2^ = 49%, *p* = 0.11). Similarly, studies employing the median as the cut-off value yielded a pooled HR of 1.50 (95% CI: 1.10–2.06; *p* = 0.01), with comparable heterogeneity (I^2^ = 49%, *p* = 0.07). Given the relatively low heterogeneity in both subgroups, a fixed-effects model was applied.

When stratified by BC subtype, miR-21 overexpression remained significantly associated with worse OS in patients with mixed subtypes of BC. In this group, the pooled HR was 2.55 (95% CI: 1.47–4.40; *p* < 0.001), and a random-effects model was used due to substantial heterogeneity (I^2^ = 77%, *p* < 0.001). A similarly strong association was observed among patients with triple-negative breast cancer, for whom high miR-21 levels were linked to poor prognosis, with a hazard ratio of 5.69 (95% CI: 3.41–9.49; *p* < 0.001). In contrast, no significant association was found in the subgroup of HER2-positive BC patients, where the pooled HR was 1.06 (95% CI: 0.54–2.10; *p* = 0.86), and heterogeneity among studies was high (I^2^ = 90%, *p* = 0.86).

Additional pre-specified subgroup analyses were conducted according to sample source (tissue vs blood), cancer stage (I–III only vs cohorts including stage IV), and ethnicity (Caucasian vs Asian). Each analysis was performed separately for OS and DFS/RFS using inverse-variance random-effects models, with χ² tests applied to assess subgroup differences. No evidence of effect modification was observed across these factors; therefore, the full forest plots and corresponding statistics are presented in the Supplementary Materials (Figures S1–S6).

### 3.6. Publication Bias and Sensitivity Analysis

**Figure 4:**
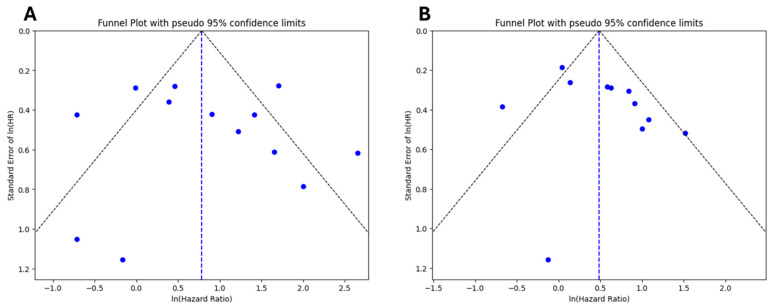
Funnel plots for publication bias assessment. (**A**) refers to OS, while (**B**) refers to DFS/RFS/DFI. Each dot represents an included study, with the x-axis showing the ln(HR) and the y-axis showing its standard error. The vertical dashed line indicates the overall pooled estimate, and the diagonal lines represent the pseudo 95% confidence limits.

The risk of publication bias was assessed using both visual inspection of funnel plot and Egger’s regression test. As shown in Figure 4A (for OS) and Figure 4B (for DFS/RFS/DFI), the funnel plots showed a degree of asymmetry, with a greater concentration of studies on the right side of the pooled effect estimate and relatively few studies on the left, particularly notable in the DFS/RFS plot. This asymmetry may indicate the presence of small-study effects or selective reporting studies with statistically significant results. Despite this visual pattern, the results of Egger’s test did not provide statistical evidence of significant publication bias. The test yielded a *p*-value of 0.699 for OS and 0.0652 for DFS/RFS, both above the conventional threshold for statistical significance. Thus, although minor asymmetry was observed in the funnel plots, there is no strong statistical indication of publication bias.

**Figure 5:**
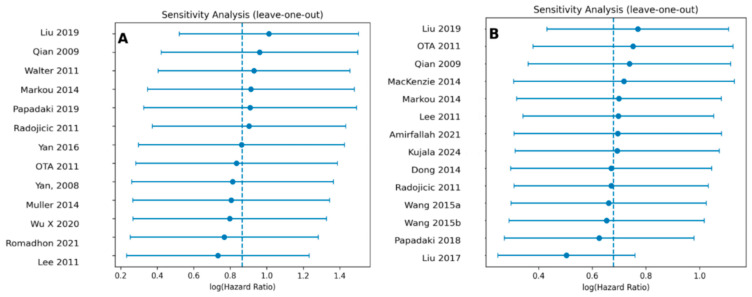
Leave-one-out sensitivity analysis plots for OS (**A**) and DFS/RFS/DFI (**B**). Each dot represents the recalculated pooled HR when the corresponding study is omitted from the meta-analysis. The vertical dashed blue line indicates the overall pooled HR [29,35,36,37,38,39,40,41,42,43,44,45,46,47,48,49,50,51,52,53].

To assess the robustness of the meta-analysis results, a leave-one-out sensitivity analysis was performed (Figure 5A,B). This analysis showed that no single study exerts a disproportionate influence on the overall hazard ratios for either OS or DFS/RFS/DFI. The pooled estimates remained consistent throughout, confirming the stability and reliability of the findings.

## 4. Discussion

Given the marked biological heterogeneity of the BC, accurate prognostic stratification is critical for guiding treatment decisions and optimizing follow-up strategies.

Among emerging biomarkers, miR-21 has garnered significant attention due to its frequent overexpression in solid tumors and its regulatory role in key oncogenic pathways [54].

In this meta-analysis, we synthesized data from 20 studies to evaluate the prognostic significance of miR-21 expression in BC. Our results demonstrate that miR-21 overexpression is significantly associated with poorer survival, both in terms of OS and recurrence-related endpoints. The pooled hazard ratio for OS was 2.37 (95% CI: 1.42–3.98; *p* = 0.001), suggesting that elevated circulating or tissue levels of miR-21 may nearly double the risk of death. These findings are consistent with previous evidence identifying miR-21 as an oncogenic miRNA (oncomiR) in multiple tumor types, including breast, lung, colorectal, and hepatocellular carcinomas [54,55]. In a 2022 systematic review, high miR-21 expression was also shown to predict reduced overall survival, with a pooled HR of 2.56 (95% CI: 1.49–4.40) [30]. Similarly, other studies have highlighted the role of miR-21 overexpression as a marker of poor prognosis in BC, demonstrating its ability to downregulate tumor suppressor genes and disrupt molecular pathways involved in tumor progression [53].

Subgroup analyses confirmed that high miR-21 expression was associated with significantly worse OS in both mixed-subtype cohorts and TNBC, a biologically aggressive subtype lacking targeted therapies. In TNBC, miR-21 appears to drive progression through multiple mechanisms, including epithelial–mesenchymal transition, increased invasiveness, and inhibition of apoptosis via suppression of tumor suppressors such as PTEN and PDCD4 [56,57,58]. These findings highlight the potential of miR-21 as a subtype-specific prognostic biomarker, especially in settings where conventional indicators may be limited.

In contrast, no significant association was observed in HER2-positive subgroups. This may reflect either genuine subtype-specific regulatory dynamics or insufficient statistical power due to the small number of HER2+ studies included. Additional data are required to draw firm conclusions in this subset.

We also evaluated how cut-off definitions for miR-21 influenced survival estimates. Both mean- and median-based thresholds yielded significant associations with OS under fixed-effect models. This consistency suggests that despite methodological variability, the negative prognostic impact of miR-21 is robust across expression quantification strategies, an important consideration for future assay standardization.

Importantly, one area of methodological homogeneity among the included studies was the analytical platform used to quantify miR-21. Sixteen of the eighteen studies employed RT-qPCR, while only two used sequencing-based approaches. This methodological consistency facilitated comparability across studies and likely reduced some technical variability, thereby supporting the reliability of pooled expression data. However, reliance on RT-qPCR also represents a limitation, as this targeted technique lacks the resolution and breadth of next-generation sequencing, which is increasingly used to profile miRNA expression at a genome-wide scale.

In the analysis of recurrence-related outcomes (DFS, DFI, RFS), the pooled HR of 1.97 (95% CI: 1.39–2.80; *p* = 0.0001) confirmed that high miR-21 levels are also associated with increased risk of disease progression or relapse, consistent with its biological function as a regulator of proliferation and apoptosis. These findings are in line with miR-21’s known roles in promoting proliferation and suppressing apoptosis [54], reinforcing its potential utility in longitudinal disease monitoring.

As expected in the meta-analyses of observational studies, we observed moderate-to-high heterogeneity (I^2^ = 77% for OS, 75% for DFS/RFS), likely driven by variability in sample types, miRNA detection methods, normalization strategies, and follow-up durations. Subgroup analyses helped mitigate some of this heterogeneity, confirming the consistency of findings across multiple contexts.

Another potential contributor to heterogeneity is the compartment-specific expression of miR-21 within the tumor microenvironment. For instance, MacKenzie et al. (2014) demonstrated that stromal fibroblast miR-21 expression may carry distinct prognostic significance compared with tumor cell expression [39]. While our meta-analysis focused exclusively on bulk tumor or circulating levels in human samples, this evidence highlights the biological complexity of miR-21 regulation and suggests that cell-type–specific analyses may refine its prognostic value in future studies.

We also assessed publication bias, which appeared low based on Egger’s test and funnel plot symmetry, although a slight asymmetry was noted in DFS/RFS studies (*p* = 0.0652). While this does not indicate significant bias statistically, the possibility of small-study effects or selective reporting cannot be entirely excluded. Importantly, leave-one-out sensitivity analyses confirmed the robustness of our findings.

Several limitations must be acknowledged. First, the number of included studies, especially in subgroup analyses, was limited, potentially reducing statistical power and the generalizability of the results. Second, although analytic methods were largely consistent, there was residual methodological heterogeneity regarding sample source and normalization strategies. Third, many studies had retrospective designs, with small cohorts, and incomplete reporting on clinical endpoints and treatment regimens, introducing a risk of bias. Finally, the predominance of RT-qPCR among included studies, while ensuring comparability, limited the availability of sequencing-based data, which could have provided broader insights into miRNA expression patterns.

## 5. Conclusions

This meta-analysis provides consistent evidence that elevated miR-21 expression is significantly associated with worse OS and DFS/RFS in patients with BC. The prognostic impact appears particularly pronounced in TNBC and mixed subtypes and remains evident across various cut-off strategies, supporting the robustness of the association while acknowledging the heterogeneity of the available studies.

Despite moderate heterogeneity and some methodological variability among studies, the overall findings support the potential role of miR-21 as a minimally invasive prognostic biomarker in BC.

Ultimately, integrating miR-21 profiling into multimodal prognostic models—alongside molecular subtype classification, gene expression panels, and clinical risk factors—may enhance personalized risk stratification and inform treatment decision-making in routine oncology practice.

Future prospective studies with standardized detection protocols, large and well-characterized cohorts, and stratification by molecular subtype, treatment modality, and disease stage are needed to validate these results and establish clinical applicability. External validation in independent populations will also be essential to confirm reproducibility.

## Figures and Tables

**Table 1:** Population-Prognostic Factors-Outcome (PFO) of the study.

Population (P)	Prognostic Factors (F)	Outcome (O)
Patients diagnosed with breast cancer	Expression of miR-21, measured in tissue, serum, plasma, or exosomes	Survival outcomes (OS, DFS, RFS, DFI)

OS: Overall Survival; DFS: Disease-Free Survival; RFS: Recurrence-Free Survival; DFI: Disease-Free Interval.

**Table 3:** Subgroup analyses of the association between circulating miR-21 expression and patient prognosis. Hazard Ratios (HR) and 95% Confidence Intervals (CI) were calculated according to different cut-off definitions (mean, median) and BC subtypes (mixed, Her2+, TNBC). Heterogeneity was assessed using the I^2^ statistic and corresponding *p*-values.

Subgroup Analysis	Number of Studies	Model	HR (95% CI)	*p* Value	Heterogeneity (I^2^, *p*-Value)
All	13	Random	2.37 (1.42–3.98)	0.001	77%, <0.001
Cut-off					
Mean	4	Fixed	4.81 (3.29–7.02)	<0.001	49%, 0.11
Median	7	Fixed	1.50 (1.10–2.06)	0.01	49%, 0.07
Type of Breast Cancer					
Mixed	9	Random	2.55 (1.47–4.40)	<0.001	77%, <0.001
Her2+	2	Fixed	1.06 (0.54–2.10)	0.86	90%, 0.86
Triple-negative	2	Fixed	5.69 (3.41–9.49)	<0.001	0%, 0.72

## Data Availability

No new data were created or analyzed in this study. Data sharing is not applicable to this article.

## Supplementary Materials

The following supporting information can be downloaded at https://www.mdpi.com/article/10.3390/ijms26199713/s1.
