# Supplementary material for: The Prognostic Power of miR-21 in Breast Cancer: A Systematic Review and Meta-Analysis
Source: Int J Mol Sci. 2025 Oct 6;26(19):9713. doi: 10.3390/ijms26199713 (PMC12524670; doi:10.3390/ijms26199713)
Supplement: Supplementary file 1 [file ijms-26-09713-s001.zip › ijms-3855672-supplementary.pdf]

Table S1. Search Strings with Boolean Operators

Database	Search String
PubMed	(((breast[Title/Abstract]) AND (miR-21[Title/Abstract] OR microRNA-21[Title/Abstract] OR has-miR-21[Title/Abstract] OR miRNA-21[Title/Abstract])) AND (cancer[Title/Abstract] OR cancers[Title/Abstract] OR carcinoma[Title/Abstract] OR carcinomas[Title/Abstract] OR neoplasm[Title/Abstract] OR neoplasms[Title/Abstract] OR tumor[Title/Abstract] OR tumors[Title/Abstract] OR tumour[Title/Abstract] OR tumours[Title/Abstract])) AND (expression[Title/Abstract] OR expressions[Title/Abstract] OR level[Title/Abstract] OR levels[Title/Abstract]
Scopus	(((breast[Title/Abstract]) AND (miR-21[Title/Abstract] OR microRNA-21[Title/Abstract] OR has-miR-21[Title/Abstract] OR miRNA-21[Title/Abstract])) AND (cancer[Title/Abstract] OR cancers[Title/Abstract] OR carcinoma[Title/Abstract] OR carcinomas[Title/Abstract] OR neoplasm[Title/Abstract] OR neoplasms[Title/Abstract] OR tumor[Title/Abstract] OR tumors[Title/Abstract] OR tumour[Title/Abstract] OR tumours[Title/Abstract])) AND (expression[Title/Abstract] OR expressions[Title/Abstract] OR level[Title/Abstract] OR levels[Title/Abstract]
Web of Science	(((TI=(breast)) AND TI=(miR-21 OR microRNA-21 OR has-miR-21 OR miRNA-21)) AND TI=(cancer OR cancers OR carcinoma OR carcinomas OR neoplasm OR neoplasms OR tumor OR tumors OR tumour OR tumours)) AND TI=(expression OR expressions OR level OR levels)

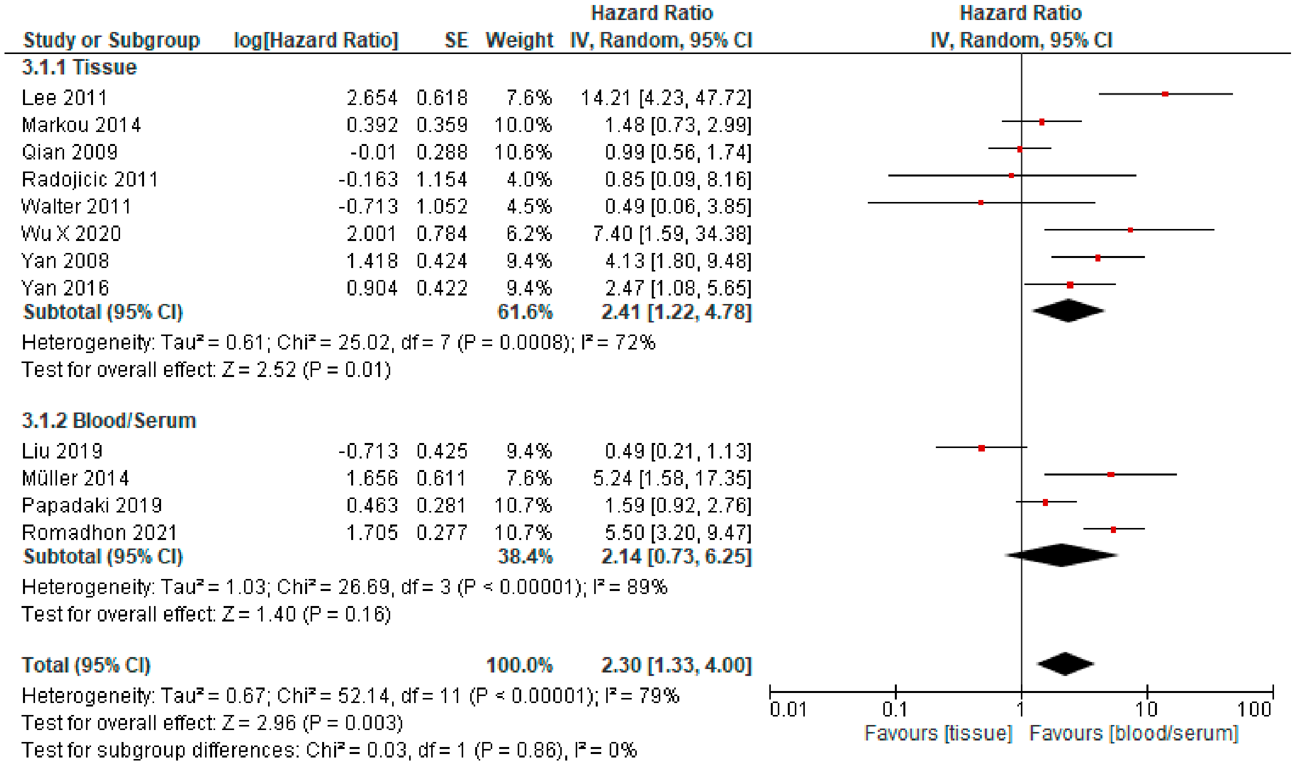

Figure S1. Overall survival (OS): Subgroup by sample source (Tissue vs Blood).

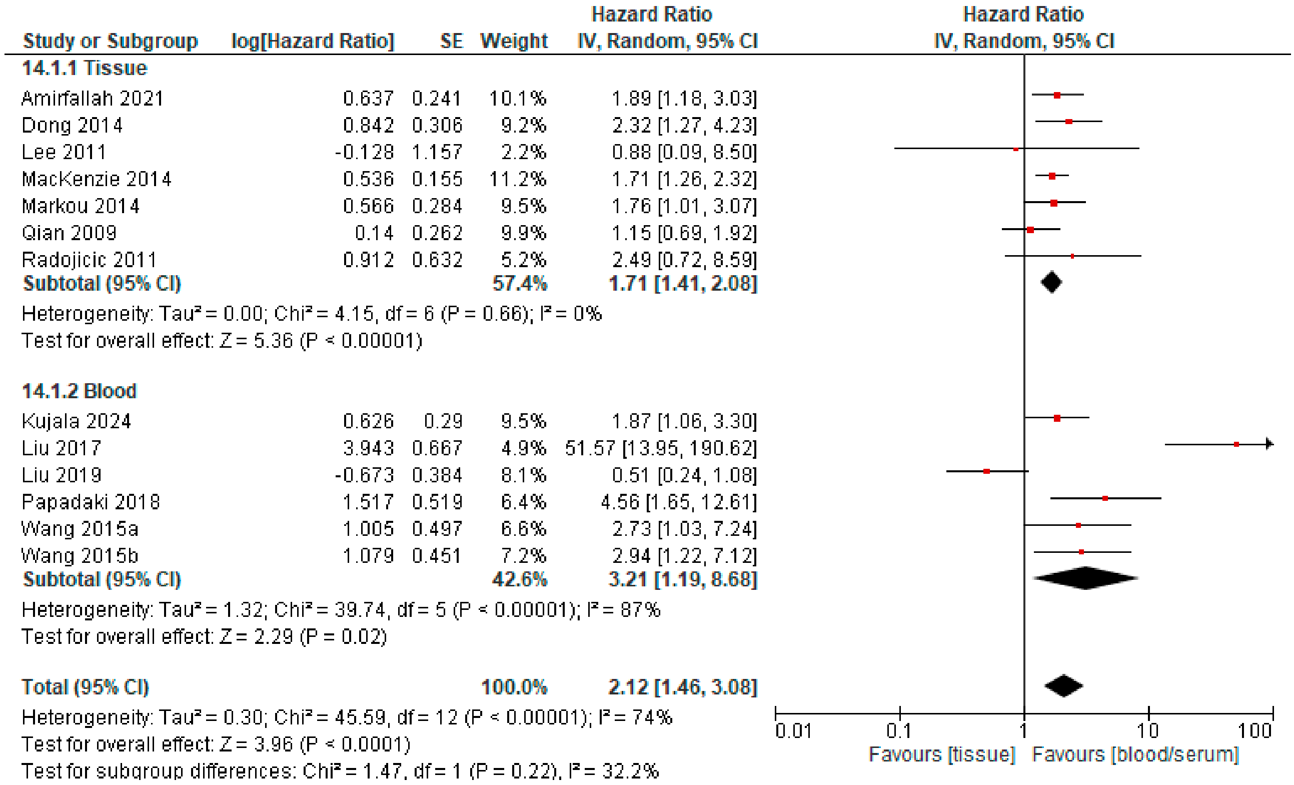

Figure S2. Disease-/Recurrence-free survival (DFS/RFS): Subgroup by sample source (Tissue vs Blood).

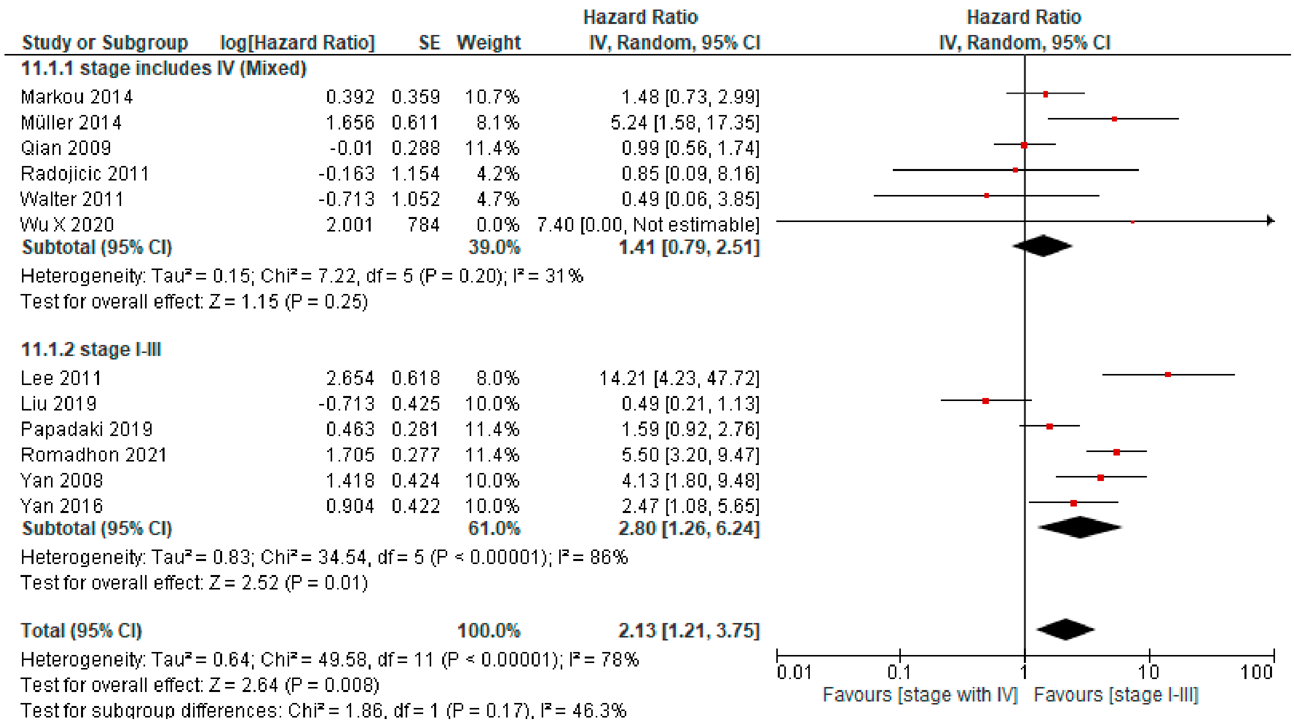

Figure S3. Overall survival (OS): Subgroup by cancer stage (I–III only vs Includes IV).

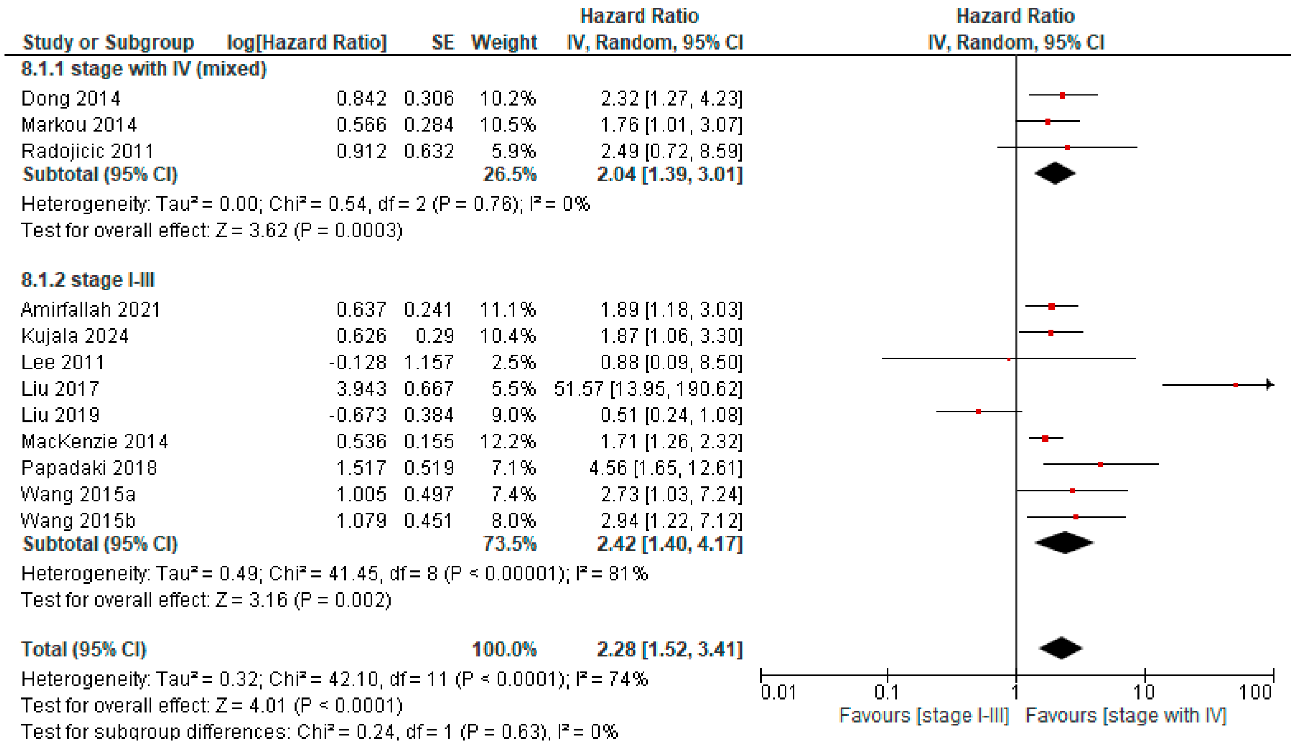

Figure S4. Disease-/Recurrence-free survival (DFS/RFS): Subgroup by cancer stage (I–III only vs Includes IV).

Study or Subgroup	log[Hazard Ratio]	SE	Weight	Hazard Ratio IV, Random, 95% CI
**12.1.1 Caucasian**				
Walter 2011	-0.713	1.052	4.0%	0.49 [0.06, 3.85]
Radojicic 2011	-0.163	1.154	3.6%	0.85 [0.09, 8.16]
Qian 2009	-0.01	0.288	9.9%	0.99 [0.56, 1.74]
Papadaki 2019	0.463	0.281	9.9%	1.59 [0.92, 2.76]
Müller 2014	1.656	0.611	7.0%	5.24 [1.58, 17.35]
Markou 2014	0.392	0.359	9.3%	1.48 [0.73, 2.99]
**Subtotal (95% CI)**			**43.6%**	**1.45 [0.93, 2.25]**
Heterogeneity: Tau² = 0.09; Chi² = 7.52, df = 5 (P = 0.18); I² = 34%				
Test for overall effect: Z = 1.63 (P = 0.10)				
**12.1.2 Asian**				
Yan 2016	0.904	0.422	8.7%	2.47 [1.08, 5.65]
Yan 2008	1.418	0.424	8.7%	4.13 [1.80, 9.48]
Wu X 2020	2.001	0.784	5.6%	7.40 [1.59, 34.38]
Romadhon 2021	1.705	0.277	9.9%	5.50 [3.20, 9.47]
OTA 2011	1.224	0.507	7.9%	3.40 [1.26, 9.19]
Liu 2019	-0.713	0.425	8.7%	0.49 [0.21, 1.13]
Lee 2011	2.654	0.618	6.9%	14.21 [4.23, 47.72]
**Subtotal (95% CI)**			**56.4%**	**3.51 [1.63, 7.54]**
Heterogeneity: Tau² = 0.82; Chi² = 30.91, df = 6 (P < 0.0001); I² = 81%				
Test for overall effect: Z = 3.22 (P = 0.001)				
**Total (95% CI)**			**100.0%**	**2.37 [1.42, 3.98]**
Heterogeneity: Tau² = 0.62; Chi² = 52.93, df = 12 (P < 0.00001); I² = 77%				
Test for overall effect: Z = 3.28 (P = 0.001)				
Test for subgroup differences: Chi² = 3.86, df = 1 (P = 0.05), I² = 74.1%				

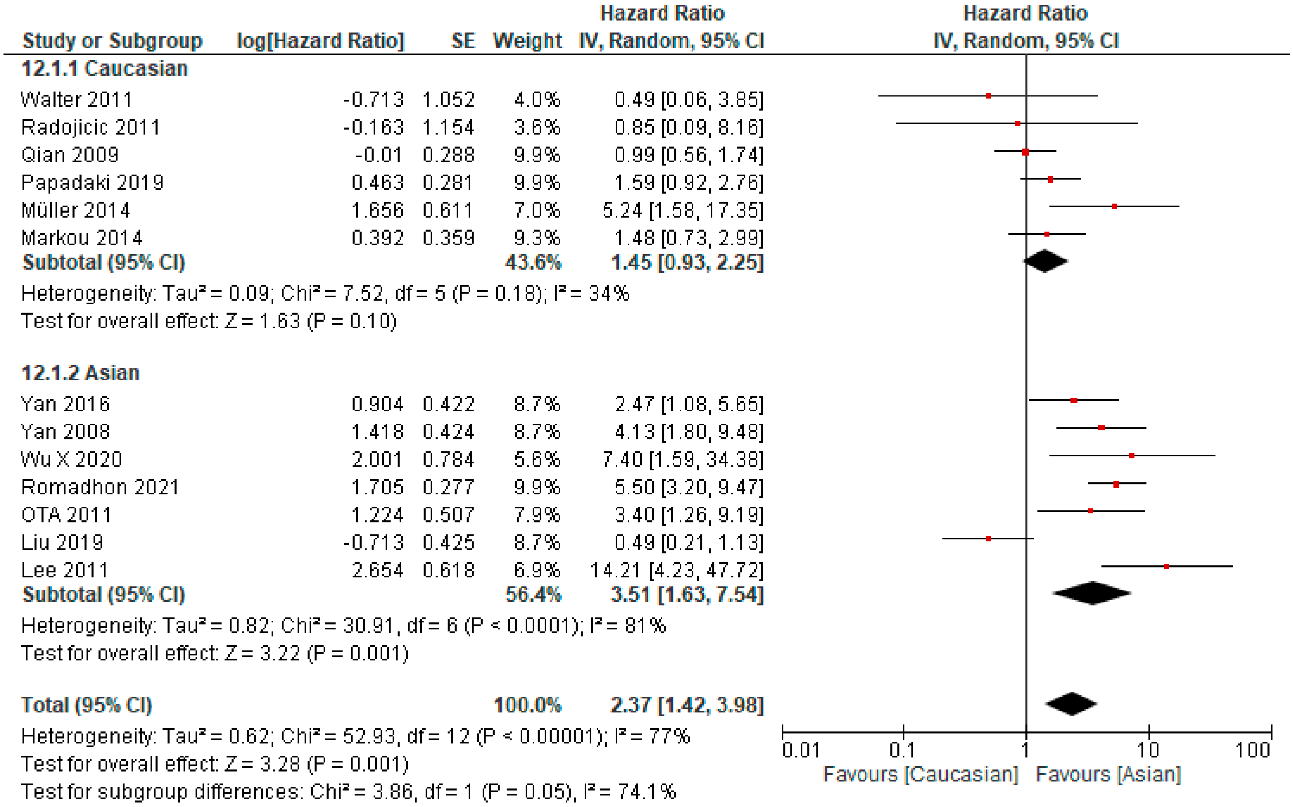

Figure S5. Overall survival (OS): Subgroup by ethnicity (Caucasian vs Asian).

Study or Subgroup	log[Hazard Ratio]	SE	Weight	Hazard Ratio IV, Random, 95% CI
**13.1.1 Caucasian**				
Amirfallah 2021	0.637	0.241	10.0%	1.89 [1.18, 3.03]
Kujala 2024	0.626	0.29	9.3%	1.87 [1.06, 3.30]
MacKenzie 2014	0.536	0.155	11.0%	1.71 [1.26, 2.32]
Markou 2014	0.566	0.284	9.4%	1.76 [1.01, 3.07]
Papadaki 2018	1.517	0.519	6.4%	4.56 [1.65, 12.61]
Radojicic 2011	0.912	0.632	5.2%	2.49 [0.72, 8.59]
**Subtotal (95% CI)**			**51.3%**	**1.86 [1.52, 2.29]**
Heterogeneity: Tau² = 0.00; Chi² = 3.54, df = 5 (P = 0.62); I² = 0%				
Test for overall effect: Z = 5.89 (P < 0.00001)				
**13.1.2 Asian**				
Dong 2014	0.842	0.306	9.1%	2.32 [1.27, 4.23]
Lee 2011	-0.128	1.157	2.2%	0.88 [0.09, 8.50]
Liu 2017	3.943	0.667	4.9%	51.57 [13.95, 190.62]
Liu 2019	-0.673	0.384	8.0%	0.51 [0.24, 1.08]
OTA 2011	0.039	0.187	10.6%	1.04 [0.72, 1.50]
Wang 2015a	1.005	0.497	6.6%	2.73 [1.03, 7.24]
Wang 2015b	1.079	0.451	7.2%	2.94 [1.22, 7.12]
**Subtotal (95% CI)**			**48.7%**	**2.30 [1.00, 5.29]**
Heterogeneity: Tau² = 0.99; Chi² = 45.79, df = 6 (P < 0.00001); I² = 87%				
Test for overall effect: Z = 1.97 (P = 0.05)				
**Total (95% CI)**			**100.0%**	**2.09 [1.44, 3.05]**
Heterogeneity: Tau² = 0.31; Chi² = 50.95, df = 12 (P < 0.00001); I² = 76%				
Test for overall effect: Z = 3.84 (P = 0.0001)				
Test for subgroup differences: Chi² = 0.23, df = 1 (P = 0.63), I² = 0%				

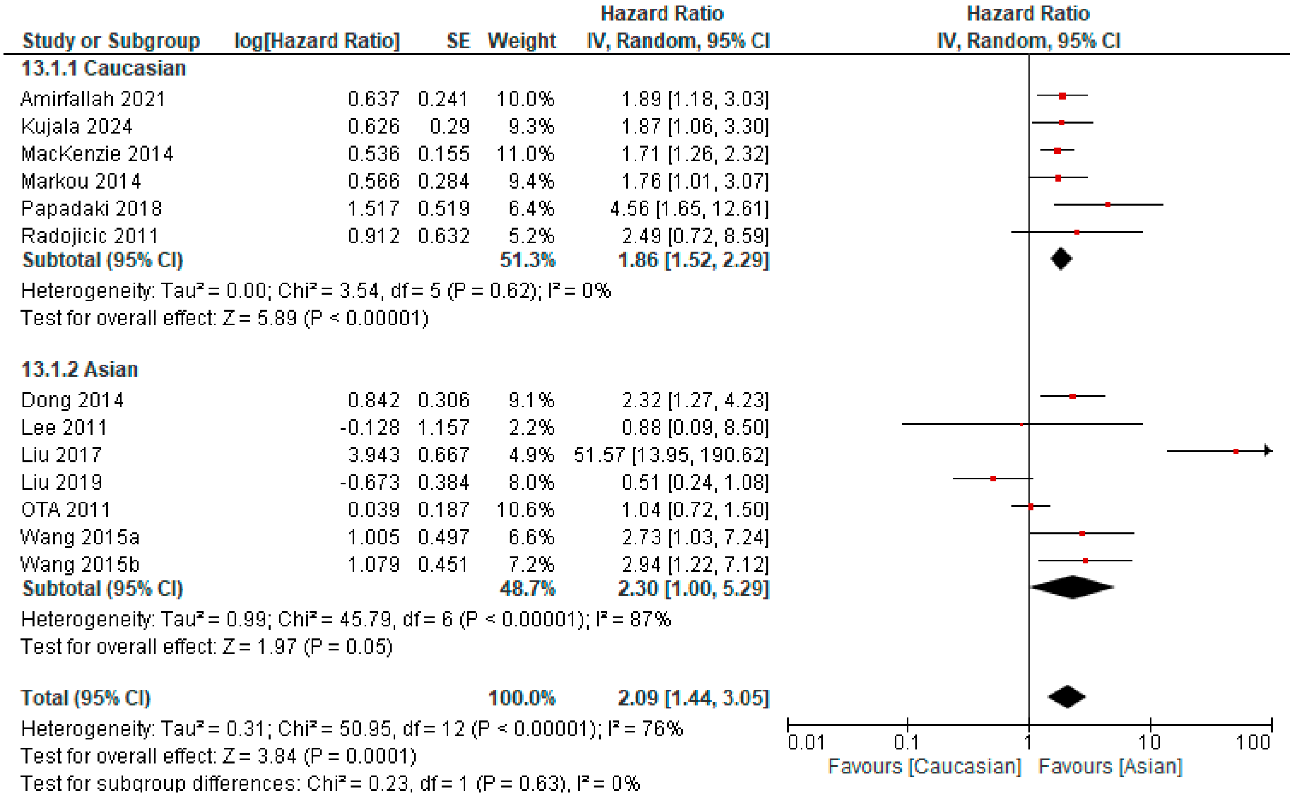

Figure S6. Disease-/Recurrence-free survival (DFS/RFS): Subgroup by ethnicity (Caucasian vs Asian).
